# Editorial: Herbal medicine for the treatment of chronic metabolic diseases, volume II

**DOI:** 10.3389/fphar.2026.1952901

**Published:** 2026-10-08

**Authors:** Yongsheng Chen

**Affiliations:** Department of Food Science and Engineering, Jinan University, Guangzhou, China

**Keywords:** chronic, health, herbal, phenolic, phytochemical

As the environment and our dietary habits undergo changes, the incidence of chronic metabolic diseases is increasing annually, which severely endangers human health and reduces quality of life. The occurrence and development of these diseases have attracted significant attention. Recently, reports have emerged suggesting that botanical materials can reduce the incidence and development of said diseases, playing a significant role in human health (Zhuang et al., 2026). For example, consuming plant-based products, such as vegetables, fruits, and tea, was found to be a simple, convenient, economical, and effective way to prevent chronic metabolic diseases (Chen et al., 2015; Guo et al., 2026). Vitamin C and β-carotene were previously considered to play a prominent role in the prevention of chronic metabolic diseases (Tribble Diane, 1999). However, numerous reports have demonstrated that phytochemicals — secondary metabolites in plants — also play a crucial role in promoting human health (Gobbo-Neto and Lopes, 2007; Kroll et al., 2003). Phytochemicals have various functions, including antioxidant activity, immune regulation, lowering cholesterol, anti-inflammatory effects, and regulating insulin resistance, blood sugar, and blood pressure (Podloucká et al., 2025; Rao et al., 2020; Wu et al., 2025; Zhang et al., 2024).

Recently, more and more attention has been paid to the prevention and treatment of chronic metabolic diseases by exploring various approaches. The efficacy of phytochemicals has been demonstrated to play a significant role in the context of traditional Chinese medicine, providing favorable evidence for the prevention and treatment of chronic metabolic diseases (Oladipupo et al., 2025; Xu et al., 2018). However, Chinese herbal medicines contain a wide array of bioactive compounds. It is not yet fully clear which compound plays a decisive role. In addition, owing to issues related to the stability and solubility of phytochemicals, the functional components and their mechanisms of action in traditional Chinese medicine have not yet been fully elucidated. Nevertheless, extensive research on the extraction, separation, component identification, functions, and metabolic pathways of phytochemicals has enriched our understanding of the efficacy and mechanisms of plant materials (Chen et al., 2023; Zhou et al., 2013). Fully verifying the physiological effects, dose-response relationship, synergistic effects, safety, and appropriate intake of phytochemicals in the prevention and treatment of chronic metabolic diseases in humans remains a formidable challenge. Given the severity of the chronic metabolic disease epidemic, strengthening research on the prevention and treatment of these conditions through phytochemicals and promoting their use to reduce the risk of morbidity and mortality from chronic metabolic diseases and reduce the burden on society and individuals is crucial.

The Research Topic “Herbal Medicine for the Treatment of Chronic Metabolic Diseases’ highlights cutting-edge studies in this field. The central objective of this Research Topic is to demonstrate how polyphenols, flavones, and their derivatives can be integrated to better understand and enhance the biological activity of herbal medicine. The 59 articles included in this Research Topic span extraction, decoction, and therapeutic mechanisms, illustrating how phytochemicals found in herbal medicine can contribute to improving the health potential of herbal medicine-derived ingredients.

Diabetes is a typical chronic metabolic disease that can cause chronic skin wounds. One original research article reports that extracts from Polygala tenuifolia, Angelica tenuissima, and Dimocarpus longan can promote keratinocyte re-epithelialization and differentiation, along with increased collagen deposition in chronic skin wounds induced in diabetic mice. With the increase in obesity and obesity-related metabolic diseases, the demand for safer and multi-targeted therapeutic strategies also grows. One original research article evaluates Galgeun-tang, finding that it can serve as a herbal intervention for obesity and metabolic disorders. Prolonged inflammation increases the risk of developing chronic metabolic problems and severely impairs quality of life. Phytochemical-rich herbal plants have been found to have anti-inflammatory effects. Specifically, the extract from Naeso-san displays potential multi-target anti-inflammatory activity. The extract of *B. indica* was evaluated for its cardioprotective and anti-inflammatory effects *in vivo*, suppressing inflammation and apoptosis. In addition, one contribution found that adjunctive berberine therapy may improve anthropometric, hormonal, metabolic, and inflammatory profiles in women with polycystic ovary syndrome.

Complementing experimental research, this Research Topic also featured review articles on the prevention of chronic metabolic disorders. These contributions synthesize evidence regarding the efficacy and safety of formulated Chinese herbal medicines for the prevention and treatment of metabolic and related disorders, including diabetes, steatotic liver disease, chronic kidney disease, asymptomatic hyperuricemia, hyperlipidemia, and diabetic cardiomyopathy, along with their antifibrotic and renoprotective effects.

A consistent theme among the contributions to this Research Topic is the understanding that the potential health benefits of phytochemicals cannot be clearly recognized without careful analysis of their active constituents and mechanisms of action. Additionally, since traditional medicine formulations consist of combinations of various herbs, there is still considerable research to be conducted on medicinal herbal products. Future research will require more in-depth analysis of phytochemical constituents, variability and standardization of herbal preparations; characterization of active constituents; herb-drug interactions; bioactivity assessments using relevant experimental models and clinical tests; and translation of preclinical findings into clinical practice. This interdisciplinary exploration has made outstanding contributions to translating herbal materials from potential bioactive ingredients into reliable elements of therapeutic strategies, functional foods, and nutraceuticals. We hope that this Research Topic will encourage further investigation of herbal plants and stimulate the development of bioactive-based approaches to maximize the therapeutic potential of these substances.
